# Testing the Shelf Life of Mozzarella-Type Cheese Packaged with Polyurethane-Based Films with Curcumin

**DOI:** 10.3390/polym17101342

**Published:** 2025-05-14

**Authors:** David Ruiz, Larissa Tessaro, Paulo José do Amaral Sobral, Yomaira Uscátegui, Luis Eduardo Diaz, Manuel F. Valero

**Affiliations:** 1 Energy, Materials and Environment Group GEMA, School of Engineering, Universidad de La Sabana, Campus del Puente del Común, Km. 7, Autopista Norte de Bogotá, Chía 140013, Colombia; davidruga@unisabana.edu.co (D.R.); yomaira.uscategui1@unisabana.edu.co (Y.U.); 2 Department of Physical Chemistry, São Carlos Institute of Chemistry, University of São Paulo, Av Trabalhador São Carlense, 400, São Carlos 13566-590, SP, Brazil; larissa.tessaro@usp.br; 3 Department of Food Engineering, School of Animal Science and Food Engineering, University of São Paulo, Av. Duque de Caxias Norte, 225, Pirassununga 13635-900, SP, Brazil; pjsobral@usp.br; 4 Food Research Center (FoRC), University of São Paulo, Rua do Lago, 250, Semi-Industrial Building, Block C, São Paulo 05508-080, SP, Brazil; 5 Bioprospecting Research Group, School of Engineering, Universidad de La Sabana, Campus del Puente del Común, Km. 7, Autopista Norte de Bogotá, Chía 140013, Colombia; luis.diaz1@unisabana.edu.co

**Keywords:** antioxidant compounds, food packaging, food spoilage, moisture

## Abstract

Cheese ripening is a time-consuming process that can lead to spoilage and product loss. The objective of this study was to evaluate the spoilage of Mozzarella-type cheese over 14 days at 4 °C, packaged with polyurethane-based films containing curcumin as an antioxidant agent. A series of physicochemical analyses were conducted, including lipid, crude fiber, and crude protein content, as well as color measurements, weight loss, moisture content, water activity, pH, titratable acidity, total and non-protein nitrogen, proteolysis index, and cheese oxidation (measured by thiobarbituric acid reactive substances—TBARS). Additionally, microbiological tests were performed to assess mesophilic bacteria, total coliforms, fungi, and yeasts. The results indicated that the polyurethane-based packaging helped maintain the Mozzarella-type cheese’s weight by significantly reducing water loss; after 14 days, the packaged cheese reached a weight loss of approximately 3%, compared to 27% of unpackaged cheese. However, that also increased moisture retention inside the package, which accelerated Mozzarella-type cheese spoilage compared to the negative control. The moisture content of packaged cheese was maintained at approximately 42%, compared to 22% of unpackaged cheese. Furthermore, the polyurethane-based films with curcumin did not exhibit any significant antioxidant effect on the cheese. It can be concluded that these polyurethane-based films are not suitable for foods with high moisture content.

## 1. Introduction

Mozzarella and Mozzarella-type cheeses are one of the most widely consumed types of cheese worldwide, valued both for direct consumption and as an ingredient in foods such as pizza. In fact, at least 70% of the world’s Mozzarella-type cheese production is used for pizza [1]. Cheese is a rich source of protein, fat, vitamins, and minerals, all of which can change during the ripening process [1]. Cheese spoilage is a time-consuming process involving both biochemical and microbiological transformations. Among the biochemical processes, complex reactions such as glycolysis, proteolysis, and lipolysis occur. Microbiological activity, driven by enzymes, bacteria, fungi, and yeasts, also plays a crucial role [2,3,4,5,6]. This microbiological activity is induced by endogenous enzymes and those produced by microorganisms, which mainly catalyze the breakdown of fats and proteins. The microorganisms also use many cheese compounds as an energy source, accelerating the spoilage of the product [7,8]. Mesophilic aerobes, fungi, yeasts, and total coliforms are commonly used to assess cheese spoilage, and therefore were selected as microbiological indicators in this study due to their influence on this process [2,3,4,5,9]. In contrast, *Salmonella*, *E. coli*, total coliforms, and *Listeria monocytogenes* are key indicators of public health risks [2,3,4,5,9] and are, therefore, not the focus of this study. Selected bacteria or fungi serve as microbiological indicators in general.

Packaging plays a key role in extending the shelf life of cheese. It serves as a barrier between the product and the environment, preventing external contamination, reducing physical damage, and limiting the transmission of oxygen and water vapor [6]. However, most cheese packaging is made from petroleum-based plastics, which are generally non-biodegradable [10]. Nevertheless, biodegradable packaging materials derived from renewable sources have been developed, such as poly-hydroxy-alkanoates (PHA), poly-3-hydroxybutyrate (PHB), polyhydroxy-valerate (PHV), poly-hydroxy-hexanoate (PHH), gluten, polylactic acid (PLA), and others. However, these materials face challenges related to mechanical performance and production costs [11,12,13]. In this context, polyurethane based on castor oil as the main polyol could be a promising alternative for producing food packaging that is partially sourced from renewable materials while offering good mechanical performance and low raw material costs [14]. In that sense, Saral et al. [15] fabricated polyurethane with zinc nanoparticles to pack carrots. Wu et al. [16] shaped a blend film of polyurethane and PLA with silver nanoparticles. In both studies, the authors aimed to retard food decay.

Additionally, most cheese packaging consists of inert polymers without any active properties. In this regard, incorporating additives such as antioxidant compounds could help delay lipid oxidation during cheese ripening, further extending its shelf life. Curcumin, a polyphenolic compound obtained from curcuma, is known for its strong antioxidant activity, low price compared to other antioxidant compounds, and high temperature tolerance (decomposition at 190 °C) [17,18,19,20]. These advantages make it valuable for active packaging solutions.

Ruiz et al. [14] conducted a comprehensive characterization of polyurethane-based films, analyzing their chemical, mechanical, morphological, and barrier properties, as well as overall and specific migration from film to simulating media. They found that both polyurethane-based films without additives and those containing 0.5% *w*/*w* curcumin are suitable for food packaging. This study serves as a continuation of previous research [14].

To the best of the authors’ knowledge, no study has been reported on polyurethane-based films activated with curcumin to package cheese. This paper aims to test the spoilage of Mozzarella-type cheese packaged with polyurethane-based films activated with curcumin as an antioxidant agent, and to evaluate the viability for this type of application.

## 2. Materials and Methods

### 2.1. Materials

Castor oil (CO) (CAS number 8001-79-4) was obtained from Químicos Campota y Cía Ltda., Bogota, Capital District, Colombia. Polyethylene glycol (PEG) with an average molecular weight of 1000 g∙mol^−1^ was sourced from Merck, Darmstadt, Hesse, Germany (CAS number 25322-68-3). Isophorone diisocyanate (IPDI) (CAS number 4098-71-9) and curcumin (CAS number 458-37-7) were obtained from Sigma-Aldrich, Burlington, MA, USA. Sodium hydroxide (NaOH) 0.1 N standard solution (CAS number 1310-73-2) and hydrochloric acid (HCl) 0.1 N standard solution (CAS number 7647-01-0) were purchased from Neon, Suzano, SP, Brazil. All 1,1,3,3 tetraethoxypropane (TEP) (CAS number 122-31-6), 2-thiobarbituric acid (TBA) (CAS number 504-17-6), ethylenediaminetetraacetic acid (EDTA) (CAS number 60-00-4), propyl gallate (PG) (CAS number 121-79-9), boric acid (Cas number 10043-35-3), sulfuric acid (CAS number 7664-93-9), tannic acid (TA) (CAS number 1401-55-4), sodium chloride (NaCl) (CAS number 7647-14-5) reagents were purchased from Sigma-Aldrich, Burlington, MA, USA. Phenolphthalein and trichloroacetic acid (TCA) (CAS number 76-03-9) were purchased from Synth, SP, Sao Paulo, Brazil. Tekbond-793 (instantaneous adhesive) was sourced from ATB Ind. e Com. de Adesivos S/A, Embu das Artes, SP, Brazil. Diatomaceous earth (CAS number 91053-39-3) was purchased from Sigma-Aldrich, Burlington, MA, USA. Mozzarella-type cheese of the same batch was produced by the dairy plant of the Faculty of Animal Science and Food Engineering, University of São Paulo (Pirassununga-SP, 21°59′46″ S, 47°25′36′′ W, Brazil). Nutritive agar, violet red bile agar (BRBA), and dextrose agar (PDA) were purchased from Scharlau (Catalunya, Spain).

### 2.2. Synthesis of Polyurethane-Based Films

The synthesis of polyurethane-based films was performed through the prepolymer method, following the method developed by Ruiz et al. [14]. The curcumin (0.5% *w*/*w* of polyurethane weight) was mixed with castor oil in a beaker and stirred at 200 rpm to overcome the miscibility problem of curcumin. Polyethylene glycol was added to the solution, which was heated at 70 °C, with continuous stirring. The ratio of castor oil and polyethylene glycol was 9:1 (mol/mol). The isophorone diisocyanate was added at 300 rpm for five minutes. The ratio of soft (polyols) and hard segment (isocyanate) was 1:1 (mol/mol). It was prepared with 25 ± 0.1 mL of prepolymer solution per round. The prepolymer solution was poured on a glass sheet, the thickness was set at 0.5 mm with an applicator of films, and then cured in an oven at 110 °C for 12 h. The scheme of the involved reaction is shown in Ruiz et al. [14]. The main physical and functional properties of polyurethane-based films have been studied previously. These data can be found in Ruiz et al. [14].

### 2.3. Application of Polyurethane-Based Active Films as Sliced Mozzarella-Type Cheese Packaging

Mozzarella-type cheese, made from whole milk and from the same batch, was manually sliced into slices measuring 1 cm × 6.5 cm × 6.5 cm and placed in sterile packaging measuring 10 cm × 10 cm made with polyurethane or polyurethane with curcumin films. The packages were sealed with Tekbond-793 and stored in a temperature-controlled chamber (BOD TE390 Tecnal, Piracicaba, SP, Brazil) at 4 ± 0.1 °C and 20 ± 0.01% of relative humidity for 14 days. Unpackaged sliced Mozzarella-type cheese was also stored as a negative control. The conservation analysis of sliced Mozzarella-type cheese was carried out at 0, 7, and 14 days. Altogether, three treatments were studied:

(i) Unpackaged sliced Mozzarella-type cheese (NC);
(ii) Sliced Mozzarella-type cheese packaged in polyurethane-based films (PU);
(iii) Sliced Mozzarella-type cheese packaged in polyurethane-based films with curcumin (CUR).

The water vapor transmission rate (WVTR) of PU and CUR is 1.868 × 10^−7^ ± 7.496 × 10^−8^ g/Pa·s·m^2^ and 2.442 × 10^−7^ ± 9.048× 10^−8^ g/Pa·s·m^2^, respectively [14].

### 2.4. Proximate Composition Analysis of the Sliced Mozzarella-Type Cheese During Storage

The proximate composition analysis of Mozzarella-type cheese was performed on day 0, in duplicate, for determination of fats (ether extract), crude fiber, and crude protein content, according to classical methods [21,22,23,24].

### 2.5. Physicochemical Analysis of the Sliced Mozzarella-Type Cheese During Storage

#### 2.5.1. Color Measurements

Color measurements were taken immediately after opening the packaging, at three different points on the sliced Mozzarella-type cheese, at all points in the experiment, on both sides of the cheese. The color parameters (L*, a*, and b*) were obtained using a MiniScan colorimeter (MSEZ 1049, HunterLab, Reston, VA, USA) in the reflectance mode using the CIELab scale, illuminant/angle of D65/10°, and 30 mm opening, according to Luciano et al. [25]. The total color difference (ΔE*) was calculated with Equation (1), being ΔL* = L*sample − L*standard, Δa* = a*sample − a*standard, and Δb* = b*sample − b*standard [26,27,28]:

(1) $${∆E}^{*}=\sqrt{({{∆L}^{*})}^{2}+({{∆a}^{*})}^{2}+({{∆b}^{*})}^{2}}$$

#### 2.5.2. Weight Loss

The weight loss of the Mozzarella type cheese slices was calculated by using Equation (2), where W_0_ and W_t_ are the initial sample weight (day 0) and sample weight at 7 and 14 days of storage, respectively [26]:

(2) $$Weight\;loss=\left(\frac{W_{0}-W_{t}}{W_{0}}\right)\times 100$$

#### 2.5.3. Moisture Content

The moisture content (MC) was determined gravimetrically. The Mozzarella-type cheese sample (1 ± 0.05 g) was dehydrated at 105 °C until constant weight for 24 h, using a forced-air circulation drying oven (MA035, Marconi, Piracicaba, SP, Brazil), after being cooled until room temperature in a desiccator. This assay was carried out in duplicate and according to modified AOAC official method 935.42 [29]. MC was then calculated using Equation (3):

(3) $$MC\;(\%)=\frac{\left(W_{1}-W_{2}\right)}{W_{1}}\times 100$$

where W_1_ is the weight of sample (g) before drying and W_2_ is the weight of sample (g) after drying.

#### 2.5.4. Water Activity

For the water activity test, samples of sliced Mozzarella-type cheese were ground and kept in room temperature (23 ± 1 °C) for 3 h to stabilize the temperature. The samples were analyzed directly in a water activity analyzer (AquaLab CX-2, Decagon^®^, Pullman, WA, USA) at room temperature [30]. The samples were analyzed in duplicate.

#### 2.5.5. pH and Titratable Acidity

For pH analysis, 5 ± 0.05 g of sliced Mozzarella-type cheese was ground and homogenized with 50 mL of distilled water at 40 °C for 10 min. The pH was determined with a portable pH meter (PG1400, Gehaka, Sao Paulo, SP, Brazil) at 25 °C.

To determine the titratable acidity, the pH analysis samples were filtered through filter paper (Whatman n° 4). Then, 25 mL of filtrate, corresponding to 2.5 g of Mozzarella-type cheese sample, were titrated with 0.1 N NaOH standard solution using 3 drops of 1% (*w/v*) phenolphthalein, according to the slightly modified AOAC official method 920.124 [31]. The results were expressed as percentage of lactic acid (Equation (4)), where V is the volume of 0.1 N NaOH standard solution used in the titration (ml), N is the normality of NaOH standard solution (0.1 N), 0.009 is equivalent weight of lactic acid neutralized by 0.1 N NaOH standard solution, and m is the weight of sample (g) [32,33]. Both pH and titratable assays were performed in duplicate.

(4) $$\%latic\;acid=\frac{V\times N\times 0.009}{m}\times 100$$

#### 2.5.6. Total and Non-Protein Nitrogen

Non-protein nitrogen was determined through the tannic acid (TA) precipitation method. 200.0 ± 0.1 mg of Mozzarella-type cheese powder and 6.00 g of 1% (*w*/*w*) of sodium chloride were weighted in a 15 mL centrifugate tube. The solution was mixed with a vortex mixer. Then, the solution was left to stand for 15 min and mixed again briefly with a vortex mixer. Then, 3.00 g of 12% (*w*/*w*) TA solution was added to the solution, thoroughly mixed with a vortex mixer, and left to stand for 30 min. The formed precipitate was recovered from the solution by centrifuging for 15 min at 1000× *g*, and the supernatant (~6 mL) was transferred to a container for nitrogen determination through the Kjeldahl method. The percentage of non-protein nitrogen (NPN) was calculated with Equation (5) [34,35]:

(5) $$\%NPN=\frac{\left(\%N\;in\;supranatant\right)\cdot (NaCl+TA+sample)}{sample}$$

where NaCl is the sodium chloride solution in g, TA is the tannic acid solution in g, NaCl is the g of NaCl, and sample is the g used in the sample.

#### 2.5.7. Thiobarbituric Acid Reactive Substances (TBARS)

To analyze the lipoperoxidation of sliced Mozzarella-type cheese during storage, the TBARS test was carried out according to the methodology described by Sørensen & Jørgensen [36], with slight modifications. For that, 2.0 ± 0.05 g of ground, sliced Mozzarella-type cheese were homogenized with 10 mL of a solution containing 7.5% (*w/v*) TCA, 0.1% (*w*/*v*) PG, and 0.1% (*w*/*v*) EDTA using a rotor-stator homogenizer (Ultraturrax^®^ IKA T25, Labotechnik, Germany) at 10,000 rpm for 30 s [25]. The samples were filtered through filter paper (Whatmann n° 1), and 5 mL of the filtrate was mixed with 5 mL of a 0.02 M TBA solution. The samples were then incubated at 100 °C for 40 min (thermostatic bath, MA-184/20, Marconi, Piracicaba, SP, Brazil), cooled with running water, and analyzed in a UV-Vis spectrophotometer (Lambda 35, Perkin-Elmer, Waltham, MA, USA) at 532 nm. TBARS values were calculated using a standard curve constructed with TEP solution (1.9 × 10^−6^ M to 2.3 × 10^−5^ M) and expressed as mg of malondialdehyde (MDA)/kg) of sample.

### 2.6. Microbiology Tests

The count of four microbiological indicators, mesophilic aerobes bacteria, total coliforms, fungus, and yeasts, were assessed. The Mozzarella-type cheese sample was weighed (11 ± 0.1 g) and diluted in 99 mL of peptone water at 40 °C and 200 rpm. Then, a serial dilution in peptone water was carried out in 10 mL tubes, taking 1 mL of previous dissolution, to add to 9 mL of peptone water (×10^−2^). This process was repeated in serial, reaching ×10^−3^, ×10^−4^, and ×10^−6^, for days 0, 7, and 14, respectively. For sowing, 1.0 mL of solution was taken in a petri dish with the corresponding culture medium. For mesophilic aerobes, Nutritive Agar was used; for total coliforms, Violet Red Bile Agar (BRBA) was used; and for fungi and yeasts, Potato Dextrose Agar (PDA) was used. The antibiotic Chloramphenicol was used as a positive control. Then, the petri dishes were incubated at 37 °C for 48 h for bacteria and 120 h for fungi and yeasts. The count was carried out once the incubation had finished in colony-forming units (CFU) [37].

### 2.7. Statistical Analysis

The data was assessed with non-parametric statistics because they did not match the assumption of normality or homoscedasticity. The data was assessed first with the Kruskal-Wallis test to verify variable differences; when these occurred, the Mann-Whitney test was applied. The median of the samples was reported. A probability of 5% was used to establish a significant difference between medians. The statistics were performed in GraphPad Prism^®^.

## 3. Results and Discussion

### 3.1. Proximate Composition of Mozzarella-Type Cheese

Considering the ether extract and crude protein content, an average of 47.16% ± 0.16 and 40.54% ± 0.32 on a wet basis, respectively, was obtained. These values are higher than those reported in the literature for Mozzarella and Mozzarella-type cheese. The fat content in the literature varies between 15% and 26%. For instance, Francolino et al. [38] reported a fat content of 16.99% ± 3.91, Costabel et al. [39] found 23.85% ± 3.23, and Tran Do [40] recorded 15.98% ± 0.74. On the other hand, no crude fiber (0.0) was determined.

Regarding protein content, literature values range from 18% to 32%. Francolino et al. [38] reported a value of 18.62% ± 4.0, Costabel et al. [39] found 24.58% ± 1.64, Carneiro et al. [41] recorded 25.34% (standard deviation not reported), and Tran Do et al. [40] found 31.68% ± 0.74.

A possible explanation for the differences between the fat content and crude protein values reported in this study and those found in the literature is moisture content (as discussed in Section 3.2.3). Other authors have reported an inverse relationship between moisture content and both fat and crude protein [42,43]. The moisture content measured in the Mozzarella-type cheese samples was 42.8% ± 0.1 on day 0, which is slightly lower than the values reported in the literature [43]. This lower moisture content could result in a reduced dilution of protein, leading to a higher fat and protein concentration [42,43].

Other factors that could influence lipid and protein content include milk quality, protein composition, and moisture levels of milk, as well as cheese production techniques. In fact, Francolino et al. [38] emphasized the importance of standardizing Mozzarella-type cheese production, as various factors can affect its nutritional composition.

### 3.2. Physicochemical Analysis of the Sliced Mozzarella-Type Cheese During Storage

#### 3.2.1. Color Measurement

**Figure 1:**
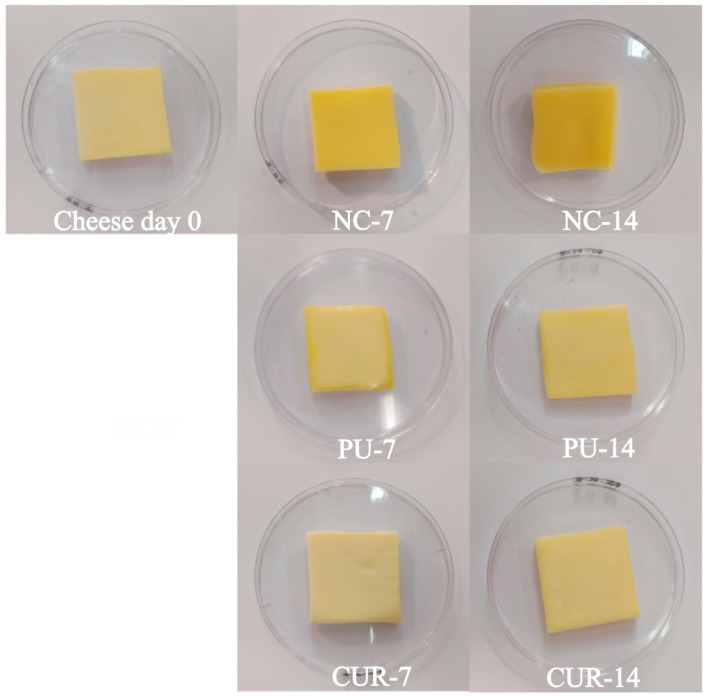
Pictures of sliced Mozzarella-type cheese after being removed from polyurethane-based films without (PU) or with 5% *w*/*w* curcumin (CUR), or unpackaged as a negative control (NC).

Food color is one of the main sensory characteristics that affect consumer acceptance [28] and can occasionally be an indication of food quality. In cheeses, color change may be related to loss of moisture, the growth of microorganisms, and lipid oxidation, for example. No statistically significant differences were observed for the color parameters among treatments, except for the a* value on side A (side up of the cheese) on day 7, where the negative control (NC) showed a statistically significant difference compared to both PU and CUR packaged treatments (Table 1). This outcome may be because Mozzarella-type cheese exhibits only minor color variations during maturation, despite the physicochemical changes occurring in the samples. Likewise, the color evolution on days 7 and 14 shows slight deterioration in color parameters, as expected [28,44] (Figure 1).

In this regard, Siroli et al. [44], in a study involving twelve Mozzarella-type cheese treatments, found statistically significant differences in L* and a* in only one treatment on day 23 of maturation. Both Siroli et al. [44] and this present study indicate that this type of cheese does not exhibit significant differences in color parameters under most treatment conditions.

#### 3.2.2. Weight Loss

**Figure 2:**
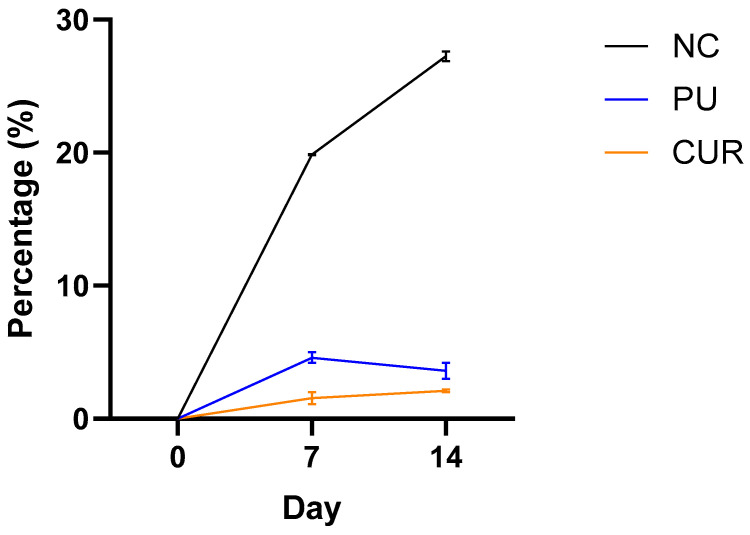
Weight loss of sliced Mozzarella-type cheese packaged with polyurethane-based films without (PU) or with 5% *w*/*w* curcumin (CUR), or unpackaged as a negative control (NC).

Packaging performs several functions that help extend the shelf life of packaged foods, one of which is protection against weight loss, mainly related to the evaporation of water from the food [6]. Packaging acts as a barrier between the food and the external environment, controlling gas exchange and water loss, preventing changes in texture, and drying out. This function performed by packaging became evident when monitoring the weight loss of sliced Mozzarella-type cheese samples (Figure 2). It highlights the differences between the NC and the two packaged sliced Mozzarella-type cheese treatments (PU and CUR). It did not find a statistically significant difference between PU and CUR packaged Mozzarella-type cheese on days 0, 7, and 14, indicating that curcumin as an additive had no observable effect on weight loss. In contrast, the NC showed a statistical difference compared to both PU and CUR on days 7 and 14. The NC exhibited remarkable weight loss on these days.

This weight loss occurred because the Mozzarella-type cheese in the NC group had no protective barrier to prevent the evaporation of water and other compounds. In contrast, the packaged Mozzarella-type cheese had a barrier that reduced weight loss due to evaporation. According to Ruiz et al. [14], the PU and CUR films exhibited very low water vapor permeability.

Franzoi et al. [45] found that moisture loss is the main factor contributing to the weight reduction of Mozzarella-type cheese, whereas protein loss had a lesser contribution. Additionally, they observed that fat, saturated fatty acids, and total solids increased in concentration as a result of moisture loss. In their study, weight loss was reported as 6.79% after 10 days and 10.96% after 21 days. These values are higher than those reported in the present study, likely because the polyurethane-based films developed in this research had a very low water vapor permeability, which reduces the moisture loss [14].

Based on the better preservation of cheese weight, it can be inferred that the packaging developed in this research may help extend the Mozzarella type cheese’s shelf life. This hypothesis will be discussed in the following results subsections.

#### 3.2.3. Moisture Content

**Figure 3:**
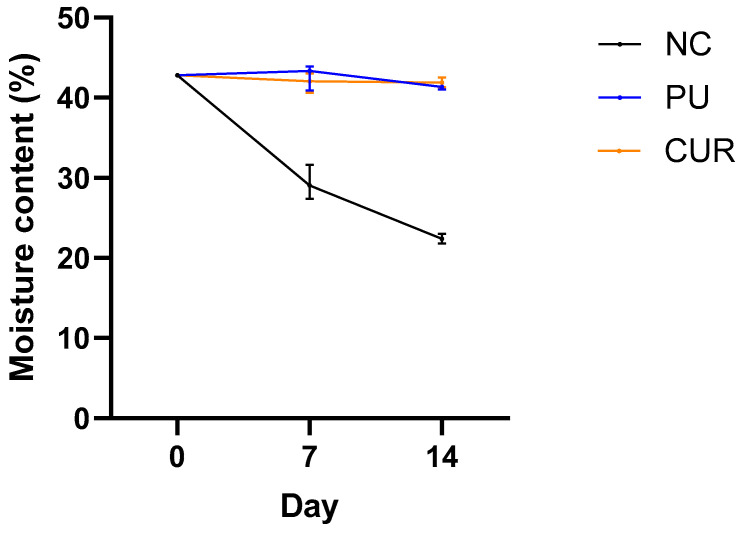
Moisture content (%) of sliced Mozzarella-type cheese packaged with polyurethane-based films without (PU) or with 5% *w*/*w* curcumin (CUR), or unpackaged as a negative control (NC).

As previously mentioned, moisture loss through evaporation is the main cause of weight loss in food. Therefore, monitoring moisture content assesses the dehydration of food over time. The packaged Mozzarella-type cheese slices showed a constant moisture content of approximately 42% during the 14 days of assay (Figure 3). No statistically significant difference was observed between PU and CUR. This stability is attributed to the polyurethane-based films’ low water vapor permeability due to their high hydrophobicity [14], which may be desirable to maintain the sensory attributes of Mozzarella-type cheese. In contrast, the NC gradually lost moisture each day, reaching 22.4% by day 14 as water and other minor compounds evaporated.

Nemati and Guimarães [46] found that Mozzarella-type cheese presented a moisture content of ~46% over nine days when packaged with high-barrier film bags. Similarly, Olivares et al. [47] reported stable moisture content values of ~49% in Mozzarella-type cheese vacuum-packaged for 57 days. These findings are consistent with the present study, where moisture content remained stable throughout the 14-day experiment.

#### 3.2.4. Water Activity

**Figure 4:**
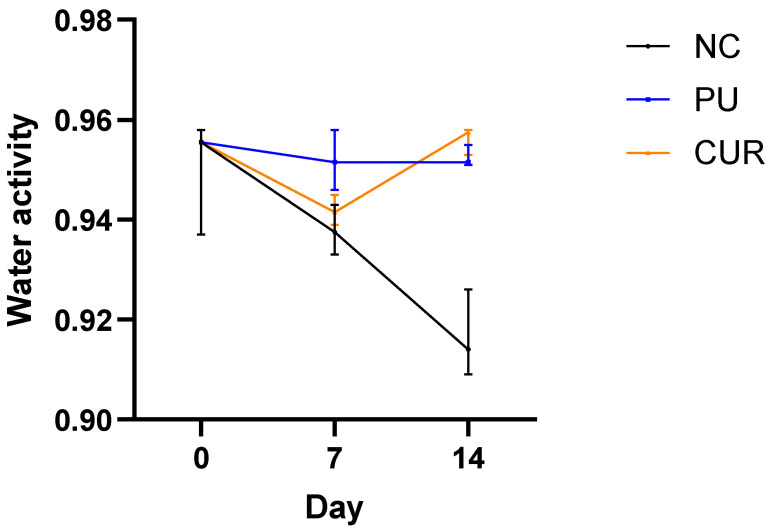
Water activity of sliced Mozzarella-type cheese packaged with polyurethane-based films without (PU) or with 5% *w*/*w* curcumin (CUR), or unpackaged as a negative control (NC).

One of the main factors responsible for food spoilage is the high water activity. Water activity is a physical-chemical parameter that measures the amount of available water in the food to facilitate chemical reactions, enzymatic activity, and microbiological growth [48]. In food in general, high water activity can facilitate lipid oxidation (values above 0.6) and the growth of fungi, yeasts, and bacteria (values above 0.8), accelerating spoilage [49]. Since Mozzarella-type cheese generally has high water activity, the water activity of packaged and unpackaged Mozzarella-type cheese was evaluated as a stability parameter (Figure 4). No statistically significant difference was observed between Mozzarella-type cheeses packaged with PU and CUR treatments on days 0 and 14. On these days, water activity remained stable at ~0.95. This high water activity indicates that a significant amount of unbound water is readily available, which is conducive to microbial proliferation [50], as shown in 3.2.8.

The stability of water activity in packaged Mozzarella-type cheese is attributed to the polyurethane-based films’ low water vapor permeability, which prevents moisture loss. Instead of escaping, the evaporated water remained inside the package. In contrast, the NC showed a significant decrease in water activity, as the evaporated moisture was released into the environment.

Zappia et al. [50] conducted a study on Mozzarella-type cheese subjected to three treatments with governing liquids and a control. They found that water activity remained between 0.96 and 0.97 over 20 days. This stability aligns with the findings of the present research, where water activity remained steady throughout the 14-day assay.

#### 3.2.5. pH and Lactic Acid

**Figure 5:**
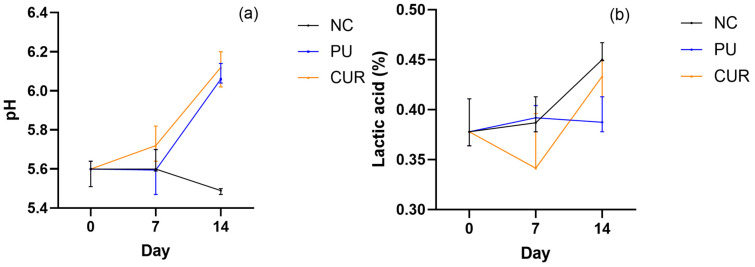
(**a**) pH and (**b**) percentage of lactic acid of sliced Mozzarella-type cheese packaged with polyurethane-based films without (PU) or with 5% *w*/*w* curcumin (CUR), or unpackaged as a negative control (NC).

The pH in cheese is a key factor that affects microbiological and enzymatic activity, lipid oxidation, taste, and other active compounds [46]. Acidity plays a crucial role in flavor development and microbial stability as well [51]. Both pH and lactic acid are linked but not necessarily proportional [52]. Mozzarella-type cheeses packaged with PU and CUR films showed similar pH values, with no statistically significative differences on any day (Figure 5a). The pH of cheese packaged with PU and CUR films increased significantly from 5.6 ± 0.13 on day 0 to 6.06 ± 0.1 and 6.12 ± 0.18 for PU and CUR, respectively, on day 14. In contrast, the pH of NC decreased to 5.49 ± 0.03, showing a statistically significant difference compared to both PU and CUR on day 14.

The increase in pH in the PU and CUR treatments could be attributed to proteolysis, which generates ammonia, as well as the metabolism of lactic acid, converting it into weaker acids or other compounds that sequester hydrogen ions [39,43,53,54]. Conversely, the decrease in pH in NC could be due to the breakdown of lactose residues by lactic acid bacteria.

The difference in pH between packaged and non-packaged Mozzarella-type cheese could be explained by the higher water activity in packaged cheese, which allows enzymatic activity and, consequently, accelerates proteolysis [4,5,50].

Nemati and Guimarães [46] found that the pH of Mozzarella-type cheese under four different manufacturing treatments remained stable or exhibited only minor changes over a nine-day shelf life assay. This result is similar to the findings of the present study, where the pH did not change significantly until day 7. However, in contrast, the pH of Mozzarella-type cheese in all treatments showed a significant change on day 14 in the present study.

On the other hand, the lactic acid levels did not show any statistical differences between treatments, except on day 14 between NC and PU (Figure 5b). Lactic acid increased considerably from day 7 to day 14 in the NC and CUR treatments. Rigueira et al. [55] reported lactic acid values ranging from 0.54 ± 0.16 on day 0 to 0.68 ± 0.23 on day 15, a trend similar to the results observed in the NC and CUR treatments. The increasing lactic acid value may be due to the growth of microorganisms breaking lactose [56].

#### 3.2.6. Total Nitrogen and Non-Protein Nitrogen

**Figure 6:**
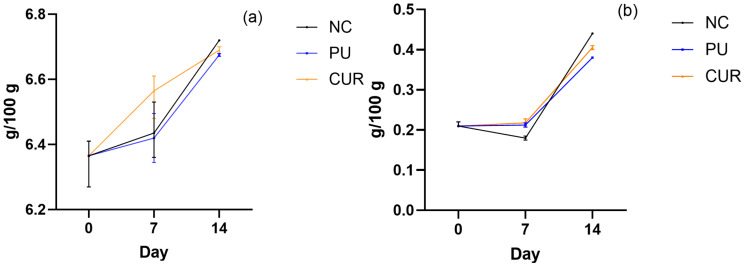
(**a**) Total nitrogen and (**b**) non-protein nitrogen of sliced Mozzarella-type cheese packaged with polyurethane-based films without (PU) or with 5% *w*/*w* curcumin (CUR), or unpackaged as a negative control (NC).

Nitrogen in dairy products exists as true protein and non-protein nitrogen. True protein nitrogen (total nitrogen) is essential for nutritional purposes. In contrast, non-protein nitrogen is often regarded as having minimal nutritional benefit and, therefore, low commercial value [57]. The total nitrogen of Mozzarella-type cheese had a notable increase on 7 and 14 days, with a statistically significant difference only on 14 days, among NC against PU and CUR (Figure 6a). The non-protein nitrogen had a steady performance on days 0 and 7 but increased notably on day 14 in all treatments (Figure 6b). A statistically significant difference was observed in NC treatment against PU and CUR treatments on days 7 and 14, as well as between PU and CUR treatments on day 14. These outcomes could imply that the Mozzarella-type cheese had a similar behavior on proteolysis due to maturation [58]. In that sense, the polyurethane-based films used for packaging did not have any significant effect on the proteolysis delay of Mozzarella-type cheese.

In the present study, the median total nitrogen increased by 0.33% ± 0.02 after 14 days. This rate is higher than that reported by El-Sayed et al. [59], who observed a mean increase of 0.35% ± 0.02 in total nitrogen over 30 days. The difference may be due to the initial total nitrogen level on day 0 of the present study, which is approximately twice that of the El-Sayed et al. [59] study. This higher initial nitrogen content could have accelerated the proteolysis rate. Additionally, El-Sayed et al. [59] attributed proteolysis to moisture content. However, in this study, we found that proteolysis showed a similar pattern across all treatments. These outcomes suggest that storage time should be considered as a key factor in the proteolysis rate, rather than moisture content. Among the factors identified by Golzarijalal et al. [60] as contributing to Mozzarella-type cheese spoilage, moisture is ranked fourth, subsequent to storage time, enzyme concentration, and calcium concentration.

Likewise, the non-protein nitrogen in the present study increased notably, from 0.21 to 0.41 g/100 g. This result differs from the Coppola et al. [61] study, where the rate of non-protein nitrogen was slightly higher. The difference could be attributed to the lower initial value in the present study compared to the Coppola et al. [61] study, which may have accelerated proteolysis in their research.

#### 3.2.7. Thiobarbituric Acid Reactive Substances (TBARS)

**Figure 7:**
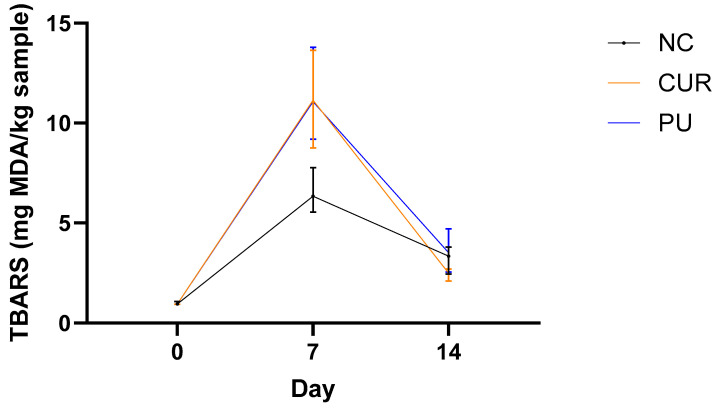
Thiobarbituric acid reactive substances (TBARS) of sliced Mozzarella-type cheese packaged with polyurethane-based films without (PU) or with 5% *w*/*w* curcumin (CUR), or unpackaged as a negative control (NC).

Lipid oxidation is one of the main causes of chemical spoilage of cheese. This affects nutritional value and sensory properties. The oxidative rate of cheese depends on the balance of antioxidant and pro-oxidant factors. The lipid oxidation is usually measured through TBARS [62]. The TBARS values of Mozzarella type cheese in this study increased significantly from day 0 to day 7 in all treatments, but the NC exhibited a smaller increase, with a statistically significant difference compared to PU and CUR (Figure 7). From day 7 to day 14, a significant reduction in lipid oxidation without any statistically significant differences among treatments was found. It highlights that curcumin as an antioxidant additive did not have any significant effect.

This trend could be explained by the fact that, on day 7, the packaged Mozzarella-type cheese with PU and CUR maintained steady moisture levels, as shown in Section 3.2.3 (Moisture Content) and Section 3.2.4 (Water Activity). The high humidity of packaged Mozzarella-type cheeses may have increased the solubility of gaseous oxygen, facilitating the lipid oxidation chain reaction [63]. The observed peak in TBARS levels in the packaged cheese on day 7 likely indicates that the cheese reached its maximum point of lipid oxidation at this time. Subsequently, on day 14, the reduction in TBARS values suggests a further progression of oxidation, leading to the formation of secondary oxidation products from the breakdown of unstable peroxides. These secondary products can include compounds such as ketones, hydrocarbons, organic acids, and aldehydes [64,65,66,67].

Taticchi et al. [68] conducted a study on two treatments of Mozzarella-type cheese stored at 4 °C for 4 days and found that TBARS values were higher on day 4. Similarly, Nemati and Guimarães [46] reported that after 9 days, the TBARS values of Mozzarella-type cheese increased progressively each day. These findings align with the present study, where TBARS’ values increased significantly until day 7. Likewise, Farbod et al. [66] found a similar trend in feta cheese, where TBARS values followed a pattern comparable to that observed in this study over a 60-day storage period.

### 3.3. Microbial Changes

**Figure 8:**
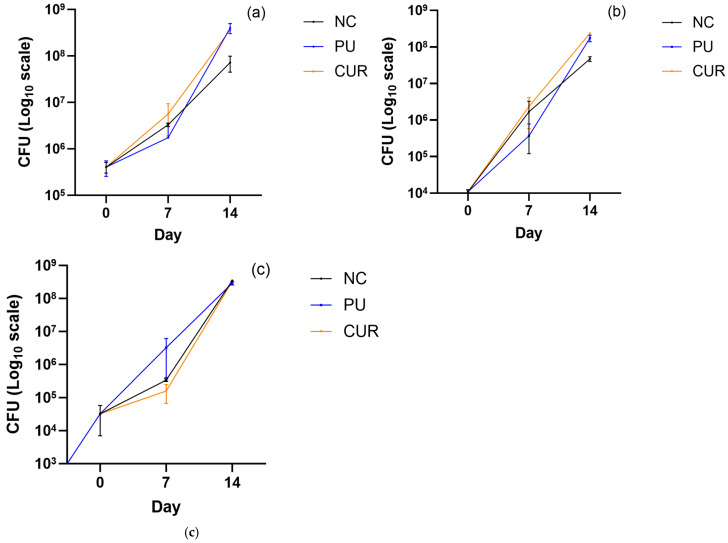
Colony-forming units of (**a**) mesophilic aerobes, (**b**) total coliforms, and (**c**) mold and yeast of sliced Mozzarella-type cheese packaged with polyurethane-based films without (PU) or with 5% *w*/*w* curcumin (CUR), or unpackaged as a negative control (NC).

According to Marrella et al. [52], “Mozzarella cheese is a complex ecosystem, characterized by a heterogenous microbial consortium”. The moisture content, availability of nutrients, and pH are key factors that influence the growth of microbiological species. The presence of microorganisms is a concern issue due to public health and spoilage microorganisms [69]. Among the spoilage microorganisms, mesophilic aerobes, total coliforms, mold, and yeasts are key microbiological indicators [70]. The four microbiological indicators—total coliforms, mold, yeast, and mesophilic aerobes—exhibited exponential growth with a similar pattern in Mozzarella-type cheese (Figure 8). However, the NC showed slower growth in colony-forming units (CFU), with a statistically significant difference observed on day 14 between NC and the packaged Mozzarella-type cheeses with PU and CUR.

Packaged Mozzarella-type cheeses exhibited a significant increase in microbiological activity, primarily due to pH and water activity, both key factors for microbial growth. A pH above 5.3 promotes microbial proliferation, while higher water activity provides a more favorable environment for microbial development [71]. Therefore, the high microbiological growth observed is likely attributed mainly to water activity, as the NC had significantly lower water activity and showed the least microbiological growth.

Marella et al. [56] conducted a study on *E. coli* in Mozzarella type cheese over a 20-day period. They found that during the first 10 days, the CFU of this bacterium increased daily, followed by a gradual decline. This outcome differs from the results of the present study, where all microbiological indicators showed a continuous increase throughout the experiment. The discrepancy may be attributed to differences in bacterial strains, despite *E. coli* being a coliform bacterium.

## 4. Conclusions

This study tested polyurethane-based films incorporated with curcumin as an antioxidant additive for packaging Mozzarella-type cheese. Three treatments were evaluated: negative control (Mozzarella-type cheese without packaging), Mozzarella-type cheese packaged with polyurethane-based film without curcumin, and Mozzarella-type cheese packaged with polyurethane-based film containing 0.5% *w*/*w* curcumin.

The polyurethane-based films helped maintain the Mozzarella-type cheese weight due to their low water vapor permeability, significantly reducing water loss through evaporation. However, this moisture retention accelerated Mozzarella-type cheese spoilage, as the increased water content inside the packaging promotes enzymatic and microbiological activity. Additionally, curcumin as an additive did not have a significant effect in reducing lipid oxidation in Mozzarella-type cheese. This suggests that reducing oxygen transmission rates may be more critical than incorporating antioxidant properties into food packaging.

Based on these findings, the polyurethane-based films developed in this study are not recommended for packaging high-moisture foods. Instead, they may be more suitable for packaging low-moisture foods or some pharmaceutical products.

## Figures and Tables

**Table 1:** Color parameters of sliced Mozzarella-type cheese packaged with polyurethane-based films without (PU) or with 5% *w*/*w* curcumin (CUR), or unpackaged as a negative control (NC).

Treatment	Color Parameter	Day 0	SD */Range	Day 7	SD */Range	Day 14	SD/Range
NC	a* side A **	1.29	0.07 ***	2.10	0.09	0.92	0.30
	b* side A	22.53	0.23	38.59	0.61	30.46	3.11
	L* side A	89.04	0.13	79.65	0.63	73.66	3.90
	a* side B	1.54	0.01	0.74	0.18	0.73	1.00
	b* side B **	23.14	0.42	27.54	0.39	31.41	2.30
	L* side B	88.75	0.59	84.81	0.54	73.01	1.46
	dE side A	21.54	0.29	39.81	0.57	35.93	4.05
	dE side B	22.22	1.17	27.38	0.85	35.93	2.9
PU	a* side A **	1.29	0.07	0.48	0.20	0.42	0.17
	b* side A	22.53	0.23	27.22	1.49	26.76	1.47
	L* side A	89.04	0.13 ***	83.87	3.56	86.39	1.36
	a* side B	1.54	0.01	0.53	0.1	0.63	0.26
	b* side B **	23.14	0.42	28.55	1.21	29.09	3.05
	L* side B	88.75	0.59	83.23	6.38	83.28	1.77
	dE side A	21.54	0.29	27.7	0.5	26.2	1.79
	dE side B	22.22	1.17	29.81	4.62	28.13	7.26
CUR	a* side A **	1.29	0.07	0.58	0.09	0.43	0.02
	b* side A	22.53	0.23	26.43	0.41	25.34	5.13
	L* side A	89.04	0.13 ***	86.16	0.4	86.23	0.9
	a* side B	1.54	0.01	1.07	1.25	0.69	0.26
	b* side B **	23.14	0.42	29.90	2.64	28.41	1.73
	L* side B	88.75	0.59	84.46	1.23	84.37	1.32
	dE side A	21.54	0.29	25.96	0.27	24.89	5.15
	dE side B	22.22	1.17	29.82	6.43	28.19	4.39

* SD: standard deviation. ** Parametric statistics. *** Statistically significant difference.

## Data Availability

The datasets generated and/or analyzed during the current study are available from the corresponding author on reasonable request.

## References

[1] Akarca G., Atik A., Atik İ., Denizkara A.J. (2023). A Comparison Study on Functional and Textural Properties of Mozzarella Cheeses Made from Bovine and Buffalo Milks Using Different Starter Cultures. Int. Dairy J..

[2] El Soda M., Awad S. (2022). Accelerated Cheese Ripening. Encyclopedia of Dairy Sciences.

[3] Klein N., Lortal S. (1999). Attenuated Starters: An Efficient Means to Influence Cheese Ripening—A Review. Int. Dairy J..

[4] Vázquez-García R., Martín-del-Campo S.T. (2023). Chapter 12—Enzyme Actions during Cheese Ripening and Production of Bioactive Compounds. Enzymes Beyond Traditional Applications in Dairy Science and Technology.

[5] Ribeiro L.R., Magalhães I.S., Tribst A.A.L., Júnior B.R.d.C.L. (2023). Chapter 7—Effects of High-Pressure Processing on Enzyme Activity in Milk and Dairy Products. Effect of High-Pressure Technologies on Enzymes.

[6] Shlush E., Davidovich-Pinhas M. (2022). Bioplastics for Food Packaging. Trends Food Sci. Technol..

[7] Garnier L., Valence F., Mounier J. (2017). Diversity and Control of Spoilage Fungi in Dairy Products: An Update. Microorganisms.

[8] Quintieri L., Caputo L., Brasca M., Fanelli F. (2021). Recent Advances in the Mechanisms and Regulation of QS in Dairy Spoilage by Pseudomonas Spp. Foods.

[9] (1997). Cheese, Mozzarella Cheese and Scarmorza Cheese [Queso. Queso Mozzarella y Queso Scarmoza].

[10] Yu Z., Rao G., Wei Y., Yu J., Wu S., Fang Y. (2019). Preparation, Characterization, and Antibacterial Properties of Biofilms Comprising Chitosan and ε-Polylysine. Int. J. Biol. Macromol..

[11] Vinod A., Sanjay M.R., Suchart S., Jyotishkumar P. (2020). Renewable and Sustainable Biobased Materials: An Assessment on Biofibers, Biofilms, Biopolymers and Biocomposites. J. Clean. Prod..

[12] Gurunathan T., Mohanty S., Nayak S.K. (2015). Isocyanate Terminated Castor Oil-Based Polyurethane Prepolymer: Synthesis and Characterization. Prog. Org. Coat..

[13] Zhong Y., Godwin P., Jin Y., Xiao H. (2020). Biodegradable Polymers and Green-Based Antimicrobial Packaging Materials: A Mini-Review. Adv. Ind. Eng. Polym. Res..

[14] Ruiz D., Uscátegui Y.L., Diaz L., Arrieta-Pérez R.R., Gómez-Tejedor J.A., Valero M.F. (2023). Obtention and Study of Polyurethane-Based Active Packaging with Curcumin and/or Chitosan Additives for Fruits and Vegetables—Part I: Analysis of Morphological, Mechanical, Barrier, and Migration Properties. Polymers.

[15] Sarojini S.K., Indumathi M.P., Rajarajeswari G.R. (2019). Mahua Oil-Based Polyurethane/Chitosan/Nano ZnO Composite Films for Biodegradable Food Packaging Applications. Int. J. Biol. Macromol..

[16] Wu Z., Zhang Z., Song X., Peng W., Zhao X., Zhao H., Liang D., Huang C., Duan Q. (2024). A Silver Nanoparticles-Polylactic Acid Microspheres/Polylactic Acid-Thermoplastic Polyurethane Nanofibers Hierarchical Antibacterial Film. Ind. Crops Prod..

[17] Mahmood K., Zia K.M., Zuber M., Salman M., Anjum M.N. (2015). Recent Developments in Curcumin and Curcumin Based Polymeric Materials for Biomedical Applications: A Review. Int. J. Biol. Macromol..

[18] Zhang T., Zhang W., Deng Y., Chu Y., Zhong Y., Wang G., Xiong Y., Liu X., Chen L., Li H. (2022). Curcumin-Based Waterborne Polyurethane-Gelatin Composite Bioactive Films for Effective UV Shielding and Inhibition of Oil Oxidation. Food Control.

[19] Lan Q., Mao X., Xia C., Zhang D., Huang P., Zhang W., Shi S., Wang Z. (2024). Curcumin Based Polyurethane Materials and Their Functional Applications: A Review. Mater. Res. Express.

[20] Chen Z., Xia Y., Liao S., Huang Y., Li Y., He Y., Tong Z., Li B. (2014). Thermal Degradation Kinetics Study of Curcumin with Nonlinear Methods. Food Chem..

[21] Silva D., de Queiroz A. (2005). Determination of crude fat or ether extracts [Determinação de gordura bruta ou do extrato etéreo]. Analysis of foods [Análise de Alimentos].

[22] Instituto Adolfo Lutz 032/IV Lipids or ether extract—Direct extraction in Soxhlet [032/IV Lipídios ou extrato etéreo—Extração direta em Soxhlet] (2005). Physico-Chemical Methods for Food Analysis [Métodos Físico-Químicos para Análise de Alimentos].

[23] Instituto Adolfo Lutz Crude fiber [044/IV Fibra bruta] (2005). Determinação da Fibra Brutas [Determinação da Fibra Bruta].

[24] Silva D., de Queiroz A. (2005). Determination of crude fiber [Determinação da fibra bruta]. Analysis of Foods [Análise de Alimentos].

[25] Luciano C.G., Tessaro L., Bonilla J., Balieiro J.C.D.C., Trindade M.A., Sobral P.J.D.A. (2022). Application of Bi-Layers Active Gelatin Films for Sliced Dried-Cured Coppa Conservation. Meat Sci..

[26] Chiabrando V., Garavaglia L., Giacalone G. (2019). The Postharvest Quality of Fresh Sweet Cherries and Strawberries with an Active Packaging System. Foods.

[27] Bonilla J., Sobral P.J.A. (2019). Gelatin-chitosan Edible Film Activated with Boldo Extract for Improving Microbiological and Antioxidant Stability of Sliced Prato Cheese. Int. J. Food Sci. Technol..

[28] Minz P.S., Saini C.S. (2021). Comparison of Computer Vision System and Colour Spectrophotometer for Colour Measurement of Mozzarella Cheese. Appl. Food Res..

[29] AOAC International Official Method 935.42, Ash of Cheese—Gravimetric Method (1995). Official Methods of Analysis of AOAC International.

[30] Troller J. (1983). Methods to Measure Water Activity. J. Food Prot..

[31] Official Method 920-124, Acidity of Cheese—Titrimetic Method (1995). Official Methods of Analysis of AOAC International.

[32] Instituto Adolfo Lutz 017/IV Determination of pH [017/IV Determinação do pH] (2005). Physico-Chemical Methods for Food Analysis [Métodos Físico-Químicos para Análise de Alimentos].

[33] Silva D., de Queiroz A. (2005). Determination of pH, titratable acidity and lactic acid of silage [Determinação do pH, da acidez titulável e do ácido láctico da silagem]. Analysis of Foods [Análise de Alimentos].

[34] Silva D., de Queiroz A. (2005). Determination of total nitrogen and crude protein [Determinação do nitrogênio total e da proteína bruta]. Analysis of Foods [Análise de Alimentos].

[35] DeVries J.W., Greene G.W., Payne A., Zbylut S., Scholl P.F., Wehling P., Evers J.M., Moore J.C. (2017). Non-Protein Nitrogen Determination: A Screening Tool for Nitrogenous Compound Adulteration of Milk Powder. Int. Dairy J..

[36] Sørensen G., Jørgensen S.S. (1996). A Critical Examination of Some Experimental Variables in the 2-Thiobarbituric Acid (TBA) Test for Lipid Oxidation in Meat Products. Z. Lebensm. Unters. Forch..

[37] (2009). Microbiology of Food and Animal Feeding Stuffs. General Requirements and Guidance for Microbiological Examinations [Microbiología de Alimentos y Productos para Alimentación Animal. Requisitos Generales y Directrices para Análisis Microbiológicos].

[38] Francolino S., Locci F., Ghiglietti R., Iezzi R., Mucchetti G. (2010). Use of Milk Protein Concentrate to Standardize Milk Composition in Italian Citric Mozzarella Cheese Making. LWT–Food Sci. Technol..

[39] Costabel L., Pauletti M.S., Hynes E. (2007). Proteolysis in Mozzarella Cheeses Manufactured by Different Industrial Processes. J. Dairy Sci..

[40] Tran Do D.H., Kong F. (2018). Texture Changes and Protein Hydrolysis in Different Cheeses under Simulated Gastric Environment. LWT.

[41] Gonçalves F.C., De Oliveira V.M., Martins F.T., Lião L.M., Ferri P.H., Queiroz Júnior L.H.K. (2022). Predicting Chemical Shelf Life of Mozzarella Cheese Submitted to Irregular Refrigeration Practices by Nuclear Magnetic Resonance Spectroscopy and Statistical Analysis. J. Food Compos. Anal..

[42] Rudan M.A., Barbano D.M., Joseph Yun J., Kindstedt P.S. (1999). Effect of Fat Reduction on Chemical Composition, Proteolysis, Functionality, and Yield of Mozzarella Cheese. J. Dairy Sci..

[43] Ah J., Tagalpallewar G.P. (2017). Functional Properties of Mozzarella Cheese for Its End Use Application. J. Food Sci. Technol..

[44] Siroli L., Patrignani F., Serrazanetti D.I., Vannini L., Salvetti E., Torriani S., Gardini F., Lanciotti R. (2016). Use of a Nisin-Producing Lactococcus Lactis Strain, Combined with Natural Antimicrobials, to Improve the Safety and Shelf-Life of Minimally Processed Sliced Apples. Food Microbiol..

[45] Franzoi M., Ghetti M., Di Monte L., De Marchi M. (2021). Investigation of Weight Loss in Mozzarella Cheese Using NIR Predicted Chemical Composition and Multivariate Analysis. J. Food Compos. Anal..

[46] Nemati V., Guimarães J.T. (2024). The Effects of Dielectric Barrier Discharge Cold Plasma on the Safety and Shelf Life Parameters of Mozzarella Cheese. Food Chem. Adv..

[47] Olivares M.L., Sihufe G.A., Capra M.L., Rubiolo A.C., Zorrilla S.E. (2012). Effect of Protective Atmospheres on Physicochemical, Microbiological and Rheological Characteristics of Sliced Mozzarella Cheese. LWT.

[48] Troller J.A., Christian J.H.B. (1978). Water Activity and Food.

[49] Barbosa-Cánovas G.V., Fontana Jr A.J., Schmidt T.P.L. (2007). Water Activity in Foods: Fundamentals and Applications.

[50] Zappia A., Branca M.L., Piscopo A., Poiana M. (2020). Shelf Life Extension of Mozzarella Cheese Packed in Preserving Liquid with Calcium Lactate and Bergamot Juice Concentrate. J. Dairy Res..

[51] Akhtar A., Araki T., Kamata T., Nei D., Khalid N. (2025). A Comparison of Low-Fat Mozzarella Cheese with Basil Seed and Taro Root Mucilage as Natural Fat Replacers through Chemical and Rheological Analysis. J. Agric. Food Res..

[52] Artur H., Mary Ann F. (2021). pH and Titratable Acidity. Cheese Making Technology e-Book.

[53] Mei J., Guo Q., Wu Y., Li Y., Yu H. (2015). Study of Proteolysis, Lipolysis, and Volatile Compounds of a Camembert-Type Cheese Manufactured Using a Freeze-Dried Tibetan Kefir Co-Culture during Ripening. Food Sci. Biotechnol..

[54] Johnson M. (2002). Cheese pH—What’s Behind the Rise and Fall?.

[55] Ubaldo J.C.S.R., Carvalho A.F., Fonseca L.M., Glória M.B.A. (2015). Bioactive Amines in Mozzarella Cheese from Milk with Varying Somatic Cell Counts. Food Chem..

[56] Marrella M., Bertani G., Ricci A., Volpe R., Roustel S., Ferriani F., Nipoti E., Neviani E., Lazzi C., Bernini V. (2022). Pseudomonas Fluorescens and Escherichia Coli in Fresh Mozzarella Cheese: Effect of Cellobiose Oxidase on Microbiological Stability during Refrigerated Shelf Life. Foods.

[57] Gowen N., Gai N., O’Mahony J.A., O’Regan J., Goulding D.A. (2025). Non-Protein Nitrogen in Dairy Ingredients: A Closer Look at Its Contribution in Infant Nutritional Product Formulation. Int. Dairy J..

[58] Ardö Y., McSweeney P.L.H., Magboul A.A.A., Upadhyay V.K., Fox P.F. (2017). Chapter 18—Biochemistry of Cheese Ripening: Proteolysis. Cheese.

[59] El-Sayed S.M., Kholif A.M.M., El-Sayed H.S., Youssef A.M. (2025). Augmenting the Quality and Shelf Life of Ras Cheese by Adding Microencapsulated Allspice Berry Extract Nanoemulsion. Food Bioprocess. Technol..

[60] Golzarijalal M., Ong L., Neoh C.R., Harvie D.J.E., Gras S.L. (2024). Machine Learning for the Prediction of Proteolysis in Mozzarella and Cheddar Cheese. Food Bioprod. Process..

[61] R C., Sorrentino E., Cinquanta L., Rossi F., Iorizzo M., L G. (1995). Shelf-Life of Mozzarella Cheese Samples Packaged without Liquid and Stored at Different Temperatures. Ital. J. Food Sci..

[62] Piscopo A., Mincione A., Summo C., Silletti R., Giacondino C., Rocco I., Pasqualone A. (2024). Influence of the Mozzarella Type on Chemical and Sensory Properties of “Pizza Margherita”. Foods.

[63] Clarke H.J., McCarthy W.P., O’Sullivan M.G., Kerry J.P., Kilcawley K.N. (2021). Oxidative Quality of Dairy Powders: Influencing Factors and Analysis. Foods.

[64] Nielsen S.S., Lipid Oxidation: Measuring Present Status (2017). Food Analysis.

[65] Flórez M., Vázquez M., Cazón P. (2023). Enhancing the Quality of Havarti Cheese: Chitosan Films with Nettle Urtica Dioica L. Extract as Slice Separators to Retard Lipid Oxidation. LWT.

[66] Farbod F., Kalbasi A., Moini S., Emam-Djomeh Z., Razavi H., Mortazavi A. (2015). Effects of Storage Time on Compositional, Micro-Structural, Rheological and Sensory Properties of Low Fat Iranian UF-Feta Cheese Fortified with Fish Oil or Fish Oil Powder. J. Food Sci. Technol..

[67] Saravani M., Ehsani A., Aliakbarlu J., Ghasempour Z. (2019). Gouda Cheese Spoilage Prevention: Biodegradable Coating Induced by *Bunium Persicum* Essential Oil and Lactoperoxidase System. Food Sci. Nutr..

[68] Taticchi A., Bartocci S., Servili M., Di Giovanni S., Pauselli M., Mourvaki E., Meo Zilio D., Terramoccia S. (2017). Effect on Quanti-Quality Milk and Mozzarella Cheese Characteristics with Further Increasing the Level of Dried Stoned Olive Pomace in Diet for Lactating Buffalo. Asian-Australas J. Anim. Sci..

[69] Losito F., Arienzo A., Bottini G., Priolisi F.R., Mari A., Antonini G. (2014). Microbiological Safety and Quality of Mozzarella Cheese Assessed by the Microbiological Survey Method. J. Dairy Sci..

[70] Akarca G., Tomar O., Gök V. (2015). Effect of Different Packaging Methods on the Quality of Stuffed and Sliced Mozzarella Cheese during Storage: Different Packaging for Sliced Mozzarella Cheese. J. Food Process. Preserv..

[71] Trmčić A., Chauhan K., Kent D.J., Ralyea R.D., Martin N.H., Boor K.J., Wiedmann M. (2016). Coliform Detection in Cheese Is Associated with Specific Cheese Characteristics, but No Association Was Found with Pathogen Detection. J. Dairy Sci..

